# Validation of Chinese version of the familiar tools use test for assessing limb apraxia in stroke patients

**DOI:** 10.3389/fneur.2025.1578179

**Published:** 2025-06-03

**Authors:** Jinni Wang, Jingxin Wei, Meilian Chen, Lu Gao, Xiaoyan Liao

**Affiliations:** ^1^School of Nursing, Southern Medical University, Guangzhou, China; ^2^Nanfang Hospital, Southern Medical University, Guangzhou, China; ^3^School of Nursing, Guangzhou Medical University, Guangzhou, China

**Keywords:** apraxia, assessment, familiar tool, stroke, validation

## Abstract

**Objective:**

To validate the Familiar Tools Use Test (FTT) of the Diagnostic Instrument for Limb Apraxia-Short Version in Chinese stroke patients.

**Methods:**

Participants were conveniently enrolled from a neurology ward in a tertiary hospital in Guangzhou, China, between April 2023 and September 2023. Internal consistency, test–retest reliability, inter-rater reliability, dimensionality, convergent validity, and divergent validity were examined.

**Results:**

In total, 110 ischemic stroke patients were included. The FTT demonstrated satisfactory internal consistency (Cronbach’s α = 0.70–0.75), test–retest reliability (ICC 0.88–0.99, 95%CI), and inter-rater reliability (Kappa coefficients ranging from 0.83 to 1.00, *p <* 0.001). Exploratory factor analysis extracted one common factor for tool selection and two common factors for action execution. There were mild to moderate correlations between the scores of the FTT scales and the MoCA (*ρ* ranged from 0.37 to 0.50), indicating satisfactory convergent and divergent validity. The moderate correlations between the scores of the FTT scales and the PTU (*ρ* ranged from 0.49 to 0.51), indicating satisfactory concurrent validity. The prevalence of limb apraxia in patients with left brain damage when selecting, producing, and executing familiar tools were 14.9, 8.5, and 8.5%, respectively. While, the prevalence of apraxia during similar tasks in those with right brain damage were 3.0, 0, and 0%, respectively.

**Conclusion:**

The FTT was reliable and valid for assessing limb apraxia among Chinese ischemic stroke patients.

## Introduction

Globally, stroke ranks as the second leading cause of mortality and the third leading cause of disability (1, 2). Ischemic stroke is the most common form of stroke, accounting for approximately 60 to 80% of all stroke cases (3). Evidence indicates that apraxia severity serves as a significant predictor of functional recovery in stroke rehabilitation (4). Apraxia is a higher-order motor cognitive disorder characterized by an impaired ability to perform skilled movements that cannot be attributed to basic sensory or motor deficits, poor comprehension, or lack of cooperation (5, 6). Limb apraxia, the most common clinical subtype (7, 8), may persist beyond the acute phase of stroke (9) and substantially compromise patients’ functional independence in activities of daily living (10). However, its impact is often overlooked and underestimated in stroke survivors (7). Therefore, early assessment of limb apraxia in stroke patients provides valuable information for healthcare professionals, enabling the formulation of targeted interventions.

The assessment of limb apraxia typically involves observing errors patients make during specific tasks such as imitation of meaningless or meaningful gestures, pantomime of tool or object use, actual tool use through familiar or novel tool settings, and execution of multistep natural actions (7). Classical tests for limb apraxia primarily focus on hand gesture imitation and pantomime tool use (6, 8, 11, 12). Deficits in pantomime of tool use are significantly associated with impaired activities of daily living (13), making affected individuals less likely to return to work than their non-apraxic counterparts (14). However, deficits in pantomime tasks may have less severe consequences than those in actual tool use. Notably, clinical impact is particularly pronounced when performance is compromised with familiar tools, which are routinely mastered for everyday activities. Apraxia involving familiar tool use is more likely to affect daily living and serves as a better predictor of stroke rehabilitation outcomes (15).

As a proxy measure for actual tool use, pantomime tasks predominantly engage conceptual knowledge systems. In contrast, actual tool use involves a closed-loop sensorimotor process that includes dynamic physical interactions among hands, tools, and target objects, providing real somatosensory feedback for online action correction and performance improvement (16). This distinction results in dissociations where patients with pantomime deficits maintain the ability to manipulate actual tools (7). Lesion-symptom mapping studies reveal a double dissociation: lesions in the ventral pathway predominantly disrupt semantic knowledge retrieval of familiar tools, while damage to the dorsal pathway impedes mechanical problem-solving in novel tool manipulation (7, 17). Assessing familiar tool use may provide unique insights into the neurocognitive architecture of praxis systems and inform the development of targeted interventions that address the integration of perceptual, semantic, and sensorimotor components in familiar tool use.

Nonetheless, few instruments incorporate actual tool use into the evaluation of limb apraxia, with the Diagnostic Instrument for Limb Apraxia (DILA-S) being a notable exception (18). The DILA-S includes both classical tasks and actual tool use tasks (17, 18), demonstrating good reliability and validity across various clinical populations (15, 19). The classic subtests of the DILA-S involve imitation of meaningful hand gestures, imitation of meaningless hand gestures, and pantomime of tool use. The distinct criteria within the DILA-S subtests allow for separate evaluations using specific cut-off points (18). Our previous study validated the classical subtests of DILA-S among Chinese stroke patients (11). The Familiar Tools Use Test (FTT) is a subtest of the DILA-S, comprising three scales that assess tool selection, action production, and execution of familiar tools (6, 18). Its German version has been validated in patients with stroke, traumatic brain injury, or dementia (19). However, it has not yet been validated in the Chinese patient population. Therefore, this study aimed to examine the psychometric properties of the FTT in Chinese ischemic stroke patients.

## Methods

Ischemic stroke patients were recruited using convenience sampling, from the neurology ward of a tertiary hospital in Guangzhou, China, between April 2023 and September 2023. Inclusion criteria were patients who: (1) clinically diagnosed with ischemic stroke, (2) stable in the condition; and (3) aged 21–80 years (as suggested by the original scale). Exclusion criteria were patients who had a: (1) previous diagnosis of epilepsy, Parkinson’s disease, or other central nervous system pathologies; (2) previous diagnosis with psychiatric disorders (such as mania, major depression, or schizophrenia) or dementia, etc., (3) refusal to participate or withdrawal from participation, and (4) inability to understand the task.

To estimate test–retest reliability, a subset of 25 patients was selected from the initial 110 post-ischemic stroke patients using convenience sampling. These patients undertook the task twice within a one-week interval. Additionally, to assess inter-rater reliability, a separate subset of 40 patients attended assessments conducted by two assessors simultaneously.

### Instruments

#### The familiar tools use test

**Figure 1:**
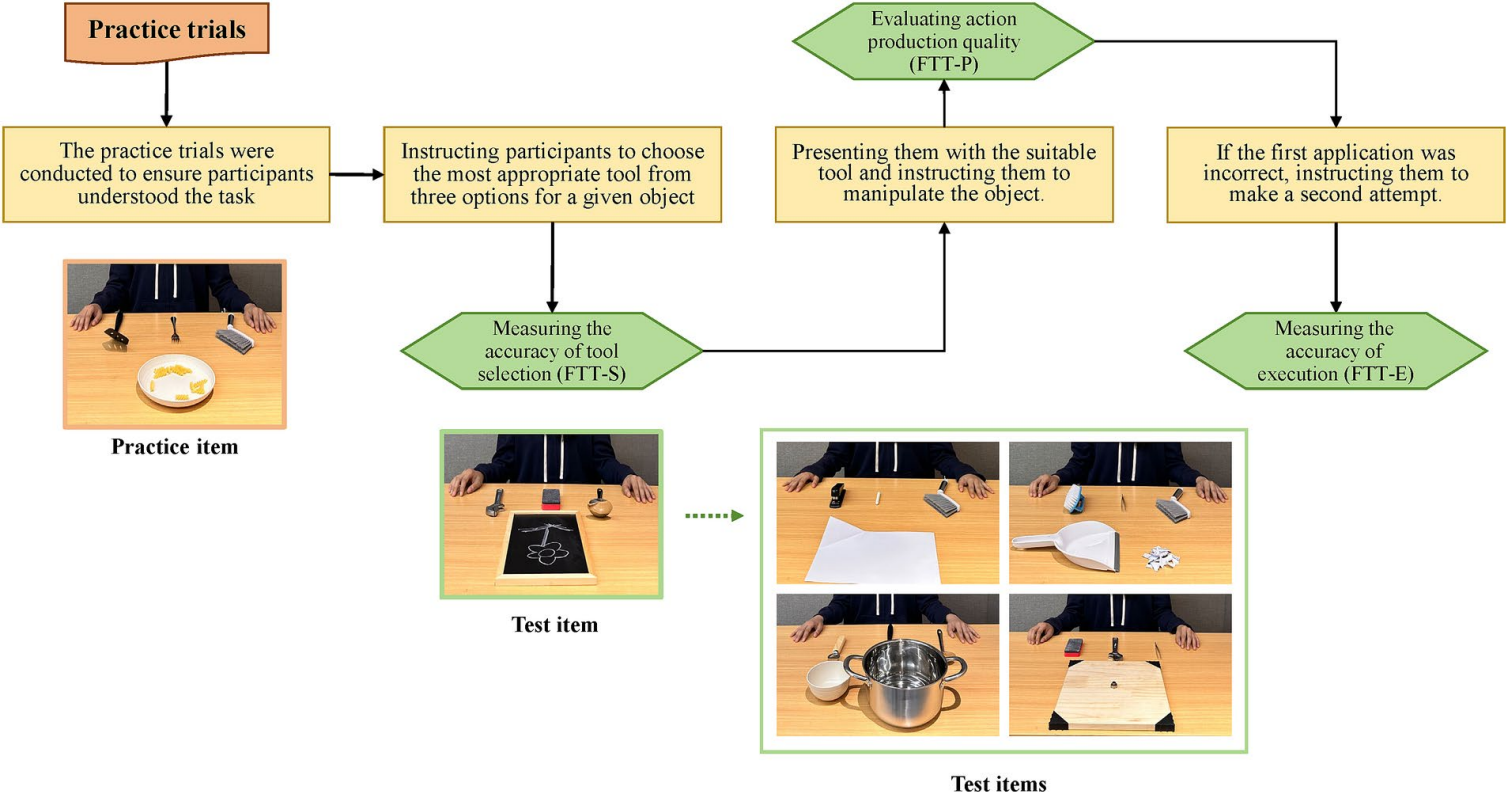
The procedures of the familiar tools use test. FTT-E, the Execution scale of the familiar tools use test; FTT-P, the Production scale of the familiar tools use test; FTT-S, the Selection scale of the familiar tools use test.

The Familiar Tools Use Test (FTT), a subtest of the DILA-S battery, assesses individuals’ performance in the actual use of familiar tools (6, 18). It consists of three practice trials and five test items. Each item presents three familiar tools (e.g., chalk, brush, or stapler) and a given object (e.g., two sheets of paper). The practice trials were conducted first to ensure participants understood the task; these trials were not scored. Subsequently, the test items were placed in front of patients sequentially. For each item, three optional tools were arranged side by side, with a target object situated behind them (from the patient’s perspective). Once the tools were properly arranged, the patient was guided through the test using standardized instructions (refer to Supplementary material 1). Tool selection was assessed by instructing participants to choose the most appropriate tool from three options for a given object (e.g., selecting from chalk, brush, or stapler to manipulate two sheets of paper). Participants’ ability to correctly apply the tool was evaluated by presenting them with the suitable tool and instructing them to manipulate the object in front of them. The FTT allows patients to be tested using the unaffected limb. If a patient demonstrated neglect, the test item should be moved to the unaffected side to ensure that the patient can perceive the object and tools. The procedures of the familiar tools use test are illustrated in Figure 1.

The FTT evaluates individuals’ performance across three scales: the selection scale (FTT-S), which measures the accuracy of tool selection; the execution scale (FTT-E), which assesses whether the action is executed correctly on the first or second attempt; and the production scale (FTT-P), which evaluates action quality based on a set of precisely defined criteria (18). On the FTT-S, choosing the correct tool on the first attempt scores 2 points per item, on the second attempt scores 1 point, and incorrect choices on both attempts score 0 points. The maximum score for the FTT-S is 10 points. The FTT-E measures the accuracy of execution on the first or second attempt, using a scoring method similar to the FTT-S. The FTT-P focuses on quality of the action production by assessing grip formation, grip orientation, movement content, and movement orientation. Grip formation involves functional grasping, such as the thumb pointing toward the functional part of the stapler; grip orientation requires the appropriate grip posture, such as a lateral or cylindrical grip; movement content involves appropriate movement, such as sliding paper between the stapler parts, pushing down, and removing the stapler; movement orientation checks spatial orientation of the tool set, such as the stapler pointing toward the paper and removing from the paper. Each appropriate criterion scores one point, yielding a maximum score of 20 points in the FTT-P. The scales are presented in Supplementary material 2.

The FTT has been validated in patients with stroke, traumatic brain injury, or dementia (19), with normative cut-off points established as less than 8 for the FTT-S, 9 for the FTT-E, and 2 for the FTT-P, for individuals aged 51–80 (18).

#### The pantomime of tool use

The Pantomime of Tool Use is a subtest of the DILA-S that has been validated in Chinese stroke patients in our prior study (11). Patients were asked to demonstrate typical gestures associated with the use of an object depicted in a picture, as if they were physically holding it. The test comprises eight items. The Production Scale (PTU-P) and the Execution Scale (PTU-E) were used to assess the pantomime gestures. The PTU-P qualitatively rated the action production of the pantomime gestures, such as grip formation, movement content, and movement orientation; each met the defined criteria scored one point, with a maximum of 24 points. Alternatively, the PTU-E scores each gesture’s correctness on the first or second attempt, awarding a “2” for a correctly executed gesture, and a “0” for two incorrect attempts, with a maximum of 16 points. The cut-off points are less than 22 for the PTU-P and less than 12 for the PTU-E, for individuals aged 51–80 (18).

#### The Montreal cognitive assessment

The Montreal Cognitive Assessment (MoCA) was used to assess general cognitive function (20). It has been validated in the Chinese population (21). The MoCA consists of 12 items across five cognitive domains: orientation, attention, memory, language, and visuospatial abilities. A maximum score of 30 can be achieved, with lower scores indicating worsening cognitive performance.

### Data collection

A trained researcher (the first author) evaluated the FTT and the pantomime of tool use. Information regarding demographics, medical history, the scores of the National Institutes of Health Stroke Scale (NIHSS), the Barthel Index (BI), and muscle strength were gathered from medical records. The muscle strength was assessed using the Medical Research Council (MRC) scale, which ranges from 0 (no muscle contraction) to 5 (normal strength). Data about lesion location and site were extracted from head MRI reports.

### Cross-cultural adaptation and pre-test

Twenty ischemic stroke patients (age 58.85 ± 10.62; female: 40%; illiterate: 10%, elementary school: 30%, junior high school: 35%, High school and above: 25%) were recruited from a tertiary hospital in Guangzhou, China, for the pre-test. They were asked to: (1) assess whether the tools in the FTT were familiar, (2) complete the related tasks; and (3) provide feedback on their understanding of the test. Pre-test results indicated that all 20 subjects found the instructions comprehensible and the tools familiar (100%), and were able to name them.

During the pretesting phase, one of the three practice items involved selecting and using a fork. All 20 patients (100%) successfully selected the fork, but 12 of them (60%) improperly attempted to use it like a spoon to scoop spiral pasta. This misuse likely arises from cultural differences, as pasta is not commonly consumed in Chinese cuisine. The expert panel opted not to replace the practice item because two alternative practice items were available and no points were awarded for practice items. Regarding test items, when presented with a task involving two sheets of paper (Item 2), 10 patients (50%) selected chalk instead of a stapler. The panel recommended using white chalk for better contrast against the white paper and ensuring the two sheets were kept separate to prevent them from being mistaken for a single sheet due to their thinness. During the test phase, 78 out of the 110 participants (70.9%) correctly selected the stapler on their first attempt, and the remaining 22 participants (20.0%) did so on their second attempt.

### Sample size calculation

The FTT comprises five test items. For factor analysis, a minimum of 100 participants is considered necessary to ensure stability of factor load (22). In examining test–retest reliability with significance level (alpha) of 0.05 and a statistical power of 80%, and assuming an intraclass correlation coefficient (ICC) of 0.5 under the alternative hypothesis, a minimum sample size of 22 is required (23). This study included 110 patients with ischemic stroke, and a subset of 25 post-ischemic stroke patients completing the FTT twice within a one-week interval.

### Ethical considerations

The Nanfang Hospital Medical Ethics Committee reviewed and approved this study (NFEC-2022-356). Prior to the study, we explained the study’s purposes, procedures, risks, and benefits to all eligible participants. Written informed consent was obtained from the participants. All personal information of the participant was kept confidential and anonymized. This study adheres to the Strengthening the Reporting of Observational Studies in Epidemiology (STROBE) guidelines for cross-sectional studies.

### Statistical analysis

Continuous variables following normal distribution were represented using mean and standard deviation, whereas those non-normally distributed were represented using medians and quartiles (Median [Q1, Q3]). Categorical variables were represented using frequencies and percentages.

Cronbach’s α was used to evaluate internal consistency while ICC was employed to assess test–retest reliability. Inter-rater reliability was quantified using Kappa coefficients, with a value above 0.8 indicating a strong agreement. Dimensionality was evaluated using exploratory factor analysis. A low Kaiser-Meyer-Olkin (KMO) value (<0.5) prompt Rasch analysis to examine the dimensionality. If the first contrast (secondary dimension) has an eigenvalue less than 3, the scale is considered probably unidimensional (24). Spearman’s rank correlation coefficients were calculated for continuous variables not following a normal distribution.

The selection of familiar tools primarily relies on retrieving functional knowledge through semantic processing, whereas their application requires additional motor-cognitive resources for action specification (17). We hypothesized a moderate to high correlation between the FTT-S scores and the MoCA scores to assess convergent validity. Additionally, we anticipated a mild correlation between the application of familiar tools (as indicated by FTT-P or FTT-E scores) and the MoCA scores to assess divergent validity. The correlations between the FTT and the Pantomime of Tool Use evaluated concurrent validity. A correlation was considered strong if the coefficient was ≥0.7, moderate if it was between 0.4 and 0.6, and weak if it was less than 0.3 (25). All data were analyzed using SPSS Version 25.0, and *p*-values less than 0.05 were considered statistically significant. Rasch analysis was conducted using WINSTEPS^®^ 4.0 (SWREG Inc., United States).

## Results

A total of 110 stroke patients completed the FTT. Among these patients, 81 (73.6%) were male, with an average age of 58.48 years (SD = 9.79). The majority, 90 (81.8%), experienced a first-onset ischemic stroke. Additionally, 98 (89.1%) were in the acute phase, while 8 (7.3%) were in the sub-acute phase (3 weeks to 6 months post-stroke onset) and 4 (3.64%) were in the chronic stage (more than 6 months). Specifically, 47 (42.7%) had left brain damage (LBD), 33 (30%) had right brain damage (RBD), and 30 (27.3%) had bilateral brain injury. The characteristics of the participants are shown in Table 1.

**Table 1:** Sociodemographic and clinical characteristics of the participants (*n* = 110).

Variables	Value
Age (mean ± SD)	58.48 ± 9.79
Sex, *n* (%)	
Male	81 (73.6)
Female	29 (26.4)
Educational level, *n* (%)	
Illiterate	5 (4.5)
Elementary school	42 (38.2)
Junior high school	36 (32.7)
High school	22 (20.0)
College	5 (4.5)
Marital status, *n* (%)	
Unmarried	2 (1.8)
Married	93 (84.5)
Divorced	8 (7.3)
Widowed	7 (6.4)
BI score (mean ± SD)	88.50 ± 17.84
BMI (mean ± SD)	23.79 ± 3.93
MRC score (mean ± SD)	
Left upper limb	4.93 ± 0.50
Right upper limb	4.70 0.96
Site of lesion, *n* (%)	
Left side	47 (42.7)
Right side	33 (30)
Bilateral	30 (27.3)
Lesion in temporal lobe, *n* (%)	17 (15.5)
First-onset ischemic stroke, *n* (%)	90 (81.8)
Time after stroke, *n* (%)	
Acute	98 (89.1)
Sub-acute	8 (7.3)
Chronic	4 (3.6)
Hospital stay duration, Median (Q1, Q3)	8 (6, 11)
NIHSS score, Median (Q1, Q3)	2 (1, 3)
MoCA score, Median (Q1, Q3)	23 (20, 26)

BMI, body mass index; BI, Barthel Index; MoCA, the Montreal Cognitive Assessment; MRC, the Medical Research Council grades for muscle strength; NIHSS, National Institutes of Health Stroke Scale.

In total, 11 (10%) exhibited deficits in tool selection, 7 (6.4%) in action production, and 7 (6.4%) in execution. The prevalence of deficits in LBD patients during selection, production, and execution of familiar tools were 14.9, 8.5, and 8.5%, respectively. In contrast, the prevalence of deficits during similar tasks in RBD patients were 3.0, 0, and 0%, respectively. The FTT scores and the prevalence are presented in Table 2.

**Table 2:** The FTT scores and the prevalence of apraxia during familiar tool use.

Scale	Scores (*n* = 110)	Apraxia, *n* (%)			
		Total (*n* = 110)	LBD (*n* = 47)	RBD (*n* = 33)	BBD (*n* = 30)
FTT-S	9.08 ± 1.56	11 (10.0)	7 (14.9)	1(3.0)	3 (10)
FTT-P	9.67 ± 1.14	7 (6.4)	4 (8.5)	0 (0.0)	3 (10)
FTT-E	19.82 ± 0.84	7 (6.4)	4 (8.5)	0 (0.0)	3 (10)

The number of whom met the cup-off point of the assessment for apraxia were displayed as *n* (%). BBD, Bilateral Brain Damage; FTT-E, the Execution scale of the familiar tools use test; FTT-P, the Production scale of the familiar tools use test; FTT-S, the Selection scale of the familiar tools use test, LBD, Left Brain Damage; RBD, Right Brain Damage.

### Internal consistency

As shown in Table 3, the Cronbach’s α for each subscale of the FTT were 0.70, 0.74, and 0.75, respectively, indicating a satisfactory internal consistency.

**Table 3:** The results of internal consistency, test–retest reliability, and intra-rater reliability.

Scale	Cronbach’s *α* (*n =* 110)	Test–retest reliability (*n =* 25)		Intra-rater reliability (*n* = 40)		
		ICC	95%CI of ICC	Kappa	*χ* ^2^	*P*
FTT-S	0.703	0.88	(0.68–0.95)	1.00	39.11	<0.001
FTT-P	0.744	0.98	(0.95–0.99)	0.83	30.01	<0.001
FTT-E	0.747	0.99	(0.98–1.00)	0.83	30.01	<0.001

CI, Confidence Interval; FTT-E, the Execution scale of the familiar tools use test; FTT-P, the Production scale of the familiar tools use test; FTT-S, the Selection scale of the familiar tools use test; ICC, Infraclass Correlation Coefficients.

### Test–retest reliability

A subset of 25 post-ischemic stroke patients completed the FTT twice within 1 week. The ICC coefficients between the two measurements for the FTT-S, FTT-E, and FTT-P scores were 0.88, 0.99, and 0.98, respectively, indicating satisfactory test–retest reliability.

### Inter-rater reliability

Two assessors scored a subset of 40 post-ischemic stroke patients simultaneously. The scores of the FTT-S, FTT-E, and FTT-P between the two assessors yielded Kappa coefficients of 1.00, 0.83, and 0.83, respectively, indicating satisfactory inter-rater reliability (Table 3).

### Dimensionality

Exploratory factor analysis extracted one common factor for the FTT-S scale and two common factors for the FTT-E scale (Table 4). The KMO values for the FTT-S, FTT-E, and FTT-P scales were 0.72, 0.63, and 0.38, respectively, suggesting that the FTT-P scores were not suitable for exploratory factor analysis. Therefore, Rasch analysis was used for testing the dimensionality of the FTT-P scores. The eigenvalues of the Rasch dimension and the first contrast (secondary dimension) were 2.98 and 1.81, respectively, suggesting that the FTT-P is probably unidimensional.

**Table 4:** The results of exploratory factor analysis for the FTT (*n =* 110).

FTT-S	Factor 1	FTT-E	Factor 1	Factor 2	FTT-P	Factor 1	Factor 2
S1	0.73	E1	0.89		P1	0.99	
S2	0.65	E2	0.87		P2	0.97	
S3	0.69	E3		0.86	P3		0.88
S4	0.63	E4		0.51	P4		0.64
S5	0.85	E5		0.88	P5		0.93
Eigenvalue	2.54		2.57	1.22		2.55	1.76
Variance explained	50.79		40.23	35.57		51.05	35.10
KMO value	0.72		0.63			0.38	
Bartlett’s test	*P* < 0.001		*P* < 0.001			*P* < 0.001	

Extraction Method: Principal Component Analysis. Rotation Method: Varimax with Kaiser Normalization.

FTT-E, the Execution scale of the familiar tools use test; FTT-P, the Production scale of the familiar tools use test; FTT-S, the Selection scale of the familiar tools use test.

### Convergent and divergent validity

As shown in Table 5, the Spearman correlation coefficient between the scores of the FTT-S and the MoCA was 0.50, indicating satisfactory convergent validity. In contrast, the Spearman correlation coefficients between the FTT-E and the MoCA (0.38), as well as between the FTT-P and the MoCA (0.37), were both below 0.4, indicating satisfactory divergent validity.

**Table 5:** The results for assessing convergent, divergent, and concurrent validity of the FTT (*n =* 110).

Scales	MoCA	PTU-P^#^	PTU-E^#^
FTT-S	0.50**^a^	0.64**	0.63**
FTT-P	0.37**^b^	0.51**	0.49**
FTT-E	0.38**^b^	0.51**	0.49**

The data were presented as Spearman correlation coefficients. FTT-E, the Execution scale of the familiar tools use test; FTT-P, the Production scale of the familiar tools use test; FTT-S, the Selection scale of the familiar tools use test; MoCA, the Montreal Cognitive Assessment; PTU-E, the Execution scale of the Pantomime of Tool Use; PTU-P, the production scale of the Pantomime of tool use. **p < 0.01. ^a^for convergent validity; ^b^for divergent validity; ^#^for concurrent validity.

### Concurrent validity

There was a significant correlation between the FTT-E and PTU-E scores (*ρ =* 0.49, *p* < 0.01). A similar correlation was observed between the FTT-P and PTU-P scores (*ρ =* 0.51, *p* < 0.01). These correlations indicate the satisfactory concurrent validity of the FTT (Table 5).

## Discussion

In this study, the psychometric properties of the FTT were examined in Chinese patients post ischemic stroke. We found that the FTT had satisfactory internal consistency, time stability, inter-rater reliability, convergent and divergent validity, and concurrent validity. To the best of our knowledge, this study is the first effort to validate a tool for measuring limb apraxia regarding familiar tool use in Chinese stroke patients.

Limb apraxia typically follows stroke or traumatic brain injury lesions, and can also occur with neurodegenerative lesions such as dementia (19). Although classical tasks like pantomime of tool use are more sensitive to limb apraxia than real tool use tasks, the latter can still significantly influence stroke patients (26). Patients suffering from limb apraxia may select the inappropriate tool (e.g., a chalk instead of a stapler to adjust two sheets of paper). Moreover, they may have problems initiating a reasonable action during tool application, though they may display alternate actions suitable for other tools (e.g., positioning a wrench perpendicular to a board for screwing), or neglect crucial steps (e.g., failing to remove the stapler from the paper after use). By applying FTT, professionals can discern the subtleties in limb apraxia, particularly noting differences between familiar tool selection and application process.

A previous lesion-symptom map study suggests that damage to the ventral regions of the temporal lobe, typically associated with functional associations, can considerably impact selection of familiar tools, while the execution of actions associated with tools is linked to lesions in the ventro-dorsal stream, particularly in the inferior parietal lobe (17). Therefore, it is proposed that the selection of familiar tools primarily depends on the retrieval of functional knowledge from semantic memory, while subsequent application of the tools might rely on further motor-cognitive resources for action specification (17). In this study, we expected to find a moderate correlation between familiar tool selection and general cognitive function, indicating convergent validity. In contrast, we anticipated only mild correlations between the application of familiar tools and general cognitive function, indicating divergent validity. Consistent with our expectations, we observed a moderate correlation between FTT-S scores and MoCA scores, along with mild correlations between FTT-P or FTT-E scores and MoCA scores. These findings support that the FTT demonstrates satisfactory convergent and divergent validity.

Our finding suggests a moderate correlation between the FTT scores, both production and execution scores, and pantomime of tool use, indicating the satisfactory concurrent validity of the FTT. The pantomime of tool use test does not account for tool selection scores. Additionally, the real tool use tasks within the FTT provide more contextual information for action opportunities (affordances), consequently reducing the demands on working memory necessary for retrieving and integrating information for action planning (17). Previous studies have noted enhanced performance when tools are applied compared to their pantomimed use (26, 27). Although these two tests possess notable differences, the existence of an overlap in inferior parietal lesion sites across action tasks raises the potential for a motor-cognitive problem in action specification (17). Two hypotheses attempt to elucidate this: the “disconnection hypothesis” posits differentiated neuropsychological processes, on the other hand, the “severity hypothesis” suggests a shared mechanism operating across a continuum of task difficulty (17).

Studies have revealed the predominant prevalence of limb apraxia in patients with left hemisphere stroke (19, 28). In this study, RBD patients performed better on the FTT than LBD patients. However, the prevalence of limb apraxia during familiar tool use in this study was obviously lower compared to a recent lesion-symptom mapping study using the same instrument, which reported higher deficits in selection (LBD: 20.7%; RBD: 2.0%), production (LBD: 37.9%; RBD: 5.9%), and execution (LBD: 48.3%; RBD: 13.7%) (17). A plausible explanation for this discrepancy may relate to the relatively mild stroke severity in the sample of this study.

To assess the dimensionality of the FTT, exploratory factor analysis was conducted in this study. However, we found that the production scores were not amenable to exploratory factor analysis, as indicated by a low KMO value, a measure verifying sampling adequacy. Consistently, a previous study suggests that 16 of 18 KMO values met the acceptable limit of 0.5 across the DILA-S subtests (17). It may be attributable to the distributional properties of the production scores, which could be non-normal due to its qualitative assessment nature of movement-content and orientation. Another contributing factor could be our relatively small sample size and the predominance of mild to moderate stroke severity in the subjects, suggesting a potential ceiling effect in production scores. Our findings using Rasch analysis suggest that the FTT-P is probably unidimensional. A larger sample size might be required for further verification.

Finally, the FTT was well accepted by patients and nurse assessors in this study, due to its simplicity and relevance to daily life. Moreover, the direct observation of subjects’ interaction with actual objects offers insights into apraxia’s impact on everyday life. Further, the FTT took only 5–15 min in this study, making it suitable for busy clinical settings. The FTT is recommended as a choice when the diagnosis of limb apraxia is made shortly after the onset of a stroke (6).

## Limitations

The study has several limitations. First, the examination interval for test–retest reliability was limited to 1 week due to the duration of hospital stay of patients with acute stroke. Second, the diagnostic accuracy of the tool was not evaluated, primarily due to the absence of a universally recognized “gold standard” for apraxia diagnosis. Third, the responsiveness of the tool was not assessed due to the constraints posed by the cross-sectional design. Additionally, the sample size was comparatively small. Future investigations should incorporate a larger, more diverse representative sample, offer a diagnostic accuracy test, and implement a cohort design for responsiveness test.

## Conclusion

The FTT is a reliable and valid tool for assessing limb apraxia in Chinese ischemic stroke patients. With its ease of use and speed, as well as its well-acceptance by subjects, it is an appropriate tool for clinical apraxia assessment.

## Data Availability

The raw data supporting the conclusions of this article will be made available by the authors, without undue reservation.
