# Supplementary material for: Validation of Chinese version of the familiar tools use test for assessing limb apraxia in stroke patients
Source: Front Neurol. 2025 Jun 3;16:1578179. doi: 10.3389/fneur.2025.1578179 (PMC12172506; doi:10.3389/fneur.2025.1578179)
Supplement: Supplementary file 1 [file Data_Sheet_1.docx]

**指导语**

“在您面前有一个日常物品（主试将手指向物品放置的位置）和三个工具（主试竖起三根手指，随后指向三个工具放置的位置）。请选择其中一个最适合与该物品一起使用的工具，把它拿在手里，演示如何使用。请实际操作一次，正确地使用，而不仅仅是做个假动作。如果您意识到您选择错了工具，可以随时更换一个。我们将从一个练习项目开始，在练习过程中，如果您有任何问题，请随时问我。”

如果第一个工具选择错误且被试没有意识到，主试以提示被试：“还有一个工具更适合与这个物品搭配使用。”

如果被试第一次使用工具错误，主试以提示被试：“您可以试试另一种方法来使用这件工具。”

**Instructions for the patient**

“I will now show you an everyday object (show where the object is set up) and three tools (show three fingers). Please select the one tool that is most suitable to be used with the object. Take it into your hand, and show how you would apply it. Please actually perform its use one time in the correct manner and not only feint it. If you realize that you have chosen the wrong tool, you can always switch to another tool. We will start with one practice item. If you have any questions, please do not hesitate to ask them.”

If the first tool is wrong and the patient does not realize that: “There is another tool that works better with this object.”

If the first application is wrong: “Maybe try another way to use the tool with this object.”
