# Supplementary material for: Validation of Chinese version of the familiar tools use test for assessing limb apraxia in stroke patients
Source: Front Neurol. 2025 Jun 3;16:1578179. doi: 10.3389/fneur.2025.1578179 (PMC12172506; doi:10.3389/fneur.2025.1578179)
Supplement: Supplementary file 2 [file Data_Sheet_2.pdf]

熟 悉 工 具 测 试

姓名：　　　　　　　　日期：

序号	条目	选　择		
0.1	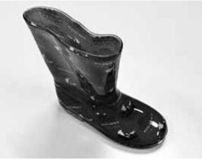<br>清洗鞋子	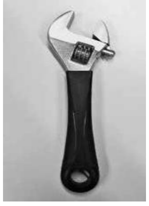	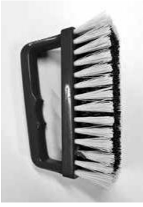	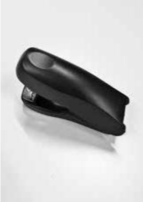
0.2	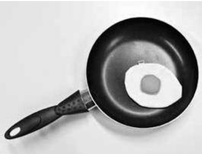<br>从锅中取出一个煎蛋	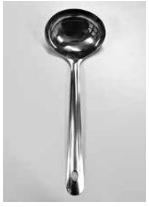	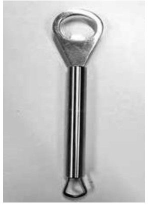	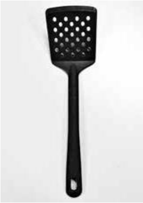
0.3	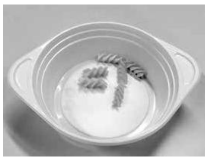<br>从盘子中取出意大利面	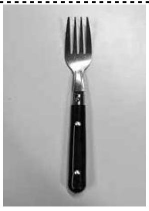	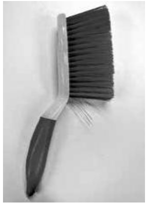	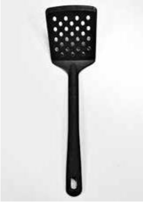
1	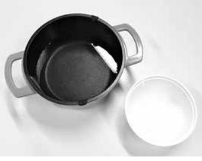<br>把汤从锅里舀到碗里	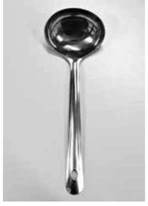	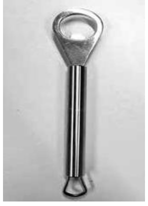	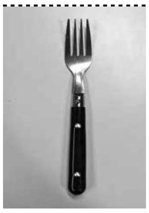
2	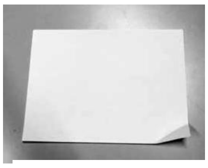<br>装订两张纸	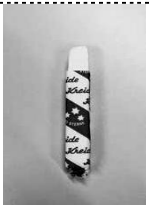	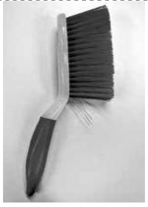	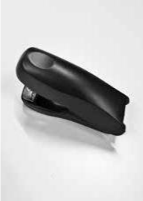
3	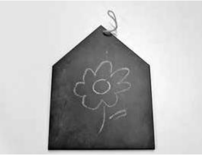<br>擦黑板	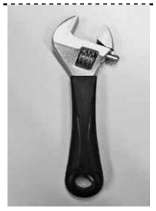	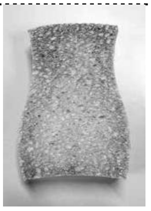	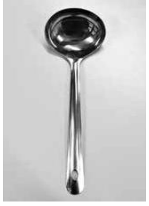
4	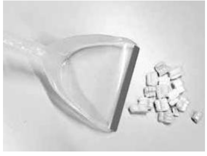<br>把垃圾扫到垃圾铲上	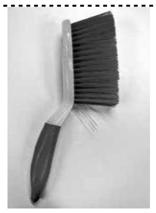	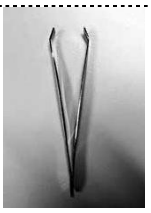	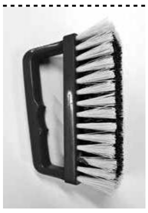
5	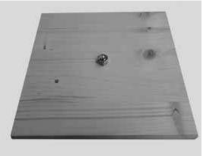<br>把螺钉拧松	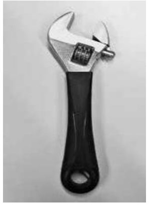	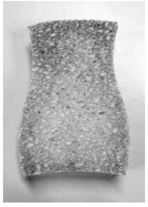	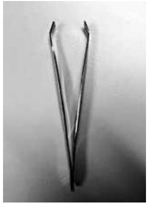

姓名：　　　　　　　　　　　　　　　　　　　　　　　日期：

选择		生 成		执行	备注
不评估		G: 指侧抓握（侧握）<br>OT: 远离参与者<br>M: 用刷子放在鞋面，重复圆周运动或刷鞋<br>O: 朝向鞋子	1<br>1<br>1<br>1	不评估	
不评估		G: 指侧抓握（侧握）或圆柱形握紧<br>OT: 朝向锅铲的功能部分<br>M: 移向煎蛋，将锅铲滑到煎蛋下，铲出煎蛋<br>O: 朝向煎蛋，铲出来（向上）	1<br>1<br>1<br>1	不评估	
不评估		G: 指侧抓握（侧握）或捏握（夹持）<br>OT: 朝向尖端<br>M: 叉向下，送入口中<br>O: 向下朝向意面，向上放入口中	1<br>1<br>1<br>1	不评估	
☐2<br>☐1<br>☐0		G: 指侧抓握（侧握）或捏握（夹持）<br>OT: 朝向长柄勺的功能部分<br>M: 舀起，转运，倾倒，<br>* =旋转前臂/手腕<br>O: 下到锅里，侧移倒向碗里	1<br>1<br>1<br><br>1	☐2<br>☐1<br>☐0	
☐2<br>☐1<br>☐0		G: 指侧抓握（侧握）或圆柱形抓握<br>OT: 朝向订书机的功能部分<br>M: 将纸张滑至订书机顶部与底部之间，向下压，将订书机从纸张上取下<br>O: 朝纸的方向移动，从纸张上移开	1<br>1<br>1<br><br>1	☐2<br>☐1<br>☐0	
☐2<br>☐1<br>☐0		G: 圆柱形松握<br>OT:远离参与者<br>M: 适用于黑板的重复旋转或擦除动作<br>O: 朝向黑板	1<br>1<br>1<br>1	☐2<br>☐1<br>☐0	
☐2<br>☐1<br>☐0		G: 圆柱形握紧或指侧抓握（侧握）<br>OT:朝向刷子的功能部分<br>M: 朝向垃圾斗的重复动作，动作来自肘部<br>O: 手刷斜向垃圾斗	1<br>1<br>1<br>1	☐2<br>☐1<br>☐0	
☐2<br>☐1<br>☐0		G: 圆柱形握紧或指侧抓握（侧握）<br>OT:朝向功能部分<br>M: 将扳手停靠在螺丝上，重复朝平行于木板的一个方向移动扳手，松开螺丝<br>O: 平行于板，朝向螺钉方向	1<br>1<br>1<br><br>1	☐2<br>☐1<br>☐0	
					总分
y	9-10		20	9-10	无失用
o	8-10				
y	8		19	8	轻度失用
o	7				
y	7		18	6-7	中度失用
o	6				
y	0-6		0-17	0-5	重度失用
o	0-5				

y = 21 - 50 岁，o = 51 - 80 岁

G = 抓握形成，OT = 抓握方向，M =运动内容，O =运动方向

选择与执行: 2 =第一次正确;1 =第二次正确;0 = 都错误
